# New Aesthetic Unit (NAU) Method: A Comprehensive Method Based on Accurate Anatomical Assessment and Precise Multilayering Panfacial Treatment for Hyaluronic Acid Fillers

**DOI:** 10.1007/s00266-024-04229-1

**Published:** 2024-07-18

**Authors:** Navid Alizadeh

**Affiliations:** Care Geneva Aesthetics, Rue Rodolphe-Toepffer 12, 1206 Geneva, Switzerland

**Keywords:** Aesthetics, Facial aging, Dermal fillers, Hyaluronic acid, Rejuvenation

## Abstract

**Background:**

Recent progress in anatomy enables a more sophisticated approach to treat patients with facial aesthetic concerns (PFAC) with HA fillers. Furthermore, advances in rheology have offered a range of HA fillers with different biomechanical properties adapted to different indications.

**Methods:**

Based on recent anatomical and rheological progresses, the author has developed a new methodology that couples an accurate patient assessment tool and a panfacial precise treatment instrument. In the presented method, the face is divided into 6 units called New Aesthetic Units (NAU). NAUs are classified on the extent of volume deficiency and asymmetry, ranging from none to moderate to severe deficiencies. After discussion with the patient regarding the assessment findings, a customized treatment plan, including timelines and number of sessions, is recommended. The modalities of the treatment are exhaustively described for each NAU based on multilayering, best practice medicine, and expert consensus available in the literature.

**Results:**

Before-and after-case studies are presented to illustrate how the NAU method is used in routine practice for the treatment of two patients with HA fillers.

**Conclusion:**

The NAU method is not only a practical and accurate roadmap for the assessment and treatment of PFAC with HA fillers, but also facilitates communication between injectors and patients and data analysis.

**Level of Evidence IV:**

This journal requires that authors assign a level of evidence to each article. For a full description of these Evidence-Based Medicine ratings, please refer to the Table of Contents or the online Instructions to Authors www.springer.com/00266.

## Introduction

Hyaluronic acid (HA) filler treatment has become the second most popular treatment in aesthetic medicine behind botulinum toxin with an average estimated growth of 8.1% per year for the next 10 years [1]. Comprehensive HA dermal filler treatments addressing the full face are increasingly recognized for delivering optimal aesthetic results by maintaining facial harmony [2]. In every other medical specialty, novice medical care practitioners are trained in a well-codified and globally accepted methodology, grounded in evidence-based medicine [3–5]. This approach equips them to assess patient pathology and propose appropriate treatment plans. However, this comprehensive methodology is notably lacking for patients seeking treatment with HA fillers, who can be collectively referred under the name “patients with facial aesthetic concerns” (PFAC). While recent tools have been introduced [6], there is an increasing need for comprehensive, systematic, precise, step by step and practical treatment methods that encompasses the face as one entity. The NAU method is a new coding system developed by the author and described here to address these gaps in treating PFACs, regardless of age, gender, or ethnicity. In this method, the face is divided in 6 different areas that we call new aesthetic units (NAU). The most important aspect of this method is its compendious anatomical foundation, which enhances assessment accuracy and facilitates teaching, as well as communication. The latter is coupled to a precise treatment tool that incorporates multilayering techniques, best medicine practices, expert opinions [7–10] and author’s experience.

## What Are NAUs?

The NAU method draws its inspiration from the anatomical units used in the treatment of burn patients [11], which are widely accepted and applied in contemporary practice, and imply that three conditions are necessary to achieve optimal aesthetic results: (1) replace a whole anatomical unit, (2) a graft or a flap of the same quality of the treated site, (3) conceal the limits of the skin graft at the natural borders of the face. Similarly, the NAU method presented here to treat PFAC meets the following criteria:

1. Consider treating a whole NAU, to have a more natural result instead of a patchy result.
2. Treat PFAC with a multilayering and composite approach. Recent remarkable advancements in the clinical anatomy of the face, including fat compartments, have significantly improved our understanding of how to address PFAC with HA fillers [12–14]. This progress enabled the implementation of a multilayering approach [8] that enhances the outcomes quality at rest and on animation by targeting different facial layers in the same treatment session. Additionally, the term “composite” refers to the use of different HA fillers with different biomechanical properties adapted to the functional attributes of the targeted layers [7, 8].
3. Concealing the boundaries between NAUs to create a natural appearance should be actively pursued [12], as it is used in burn patients.

**Fig. 1:**
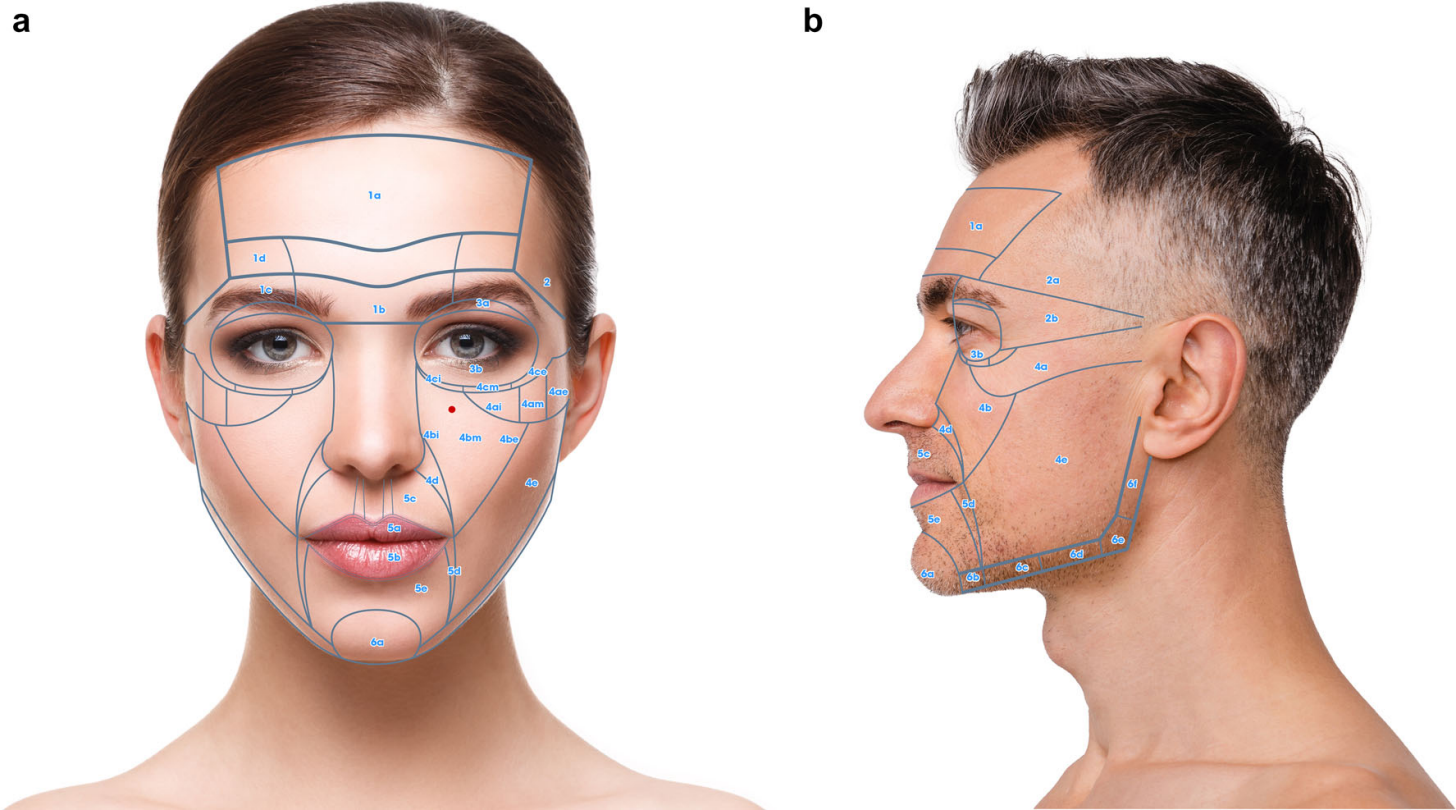
**a** Frontal view of the NAUs. **b** Profile view of the NAUs

**Fig. 2:**
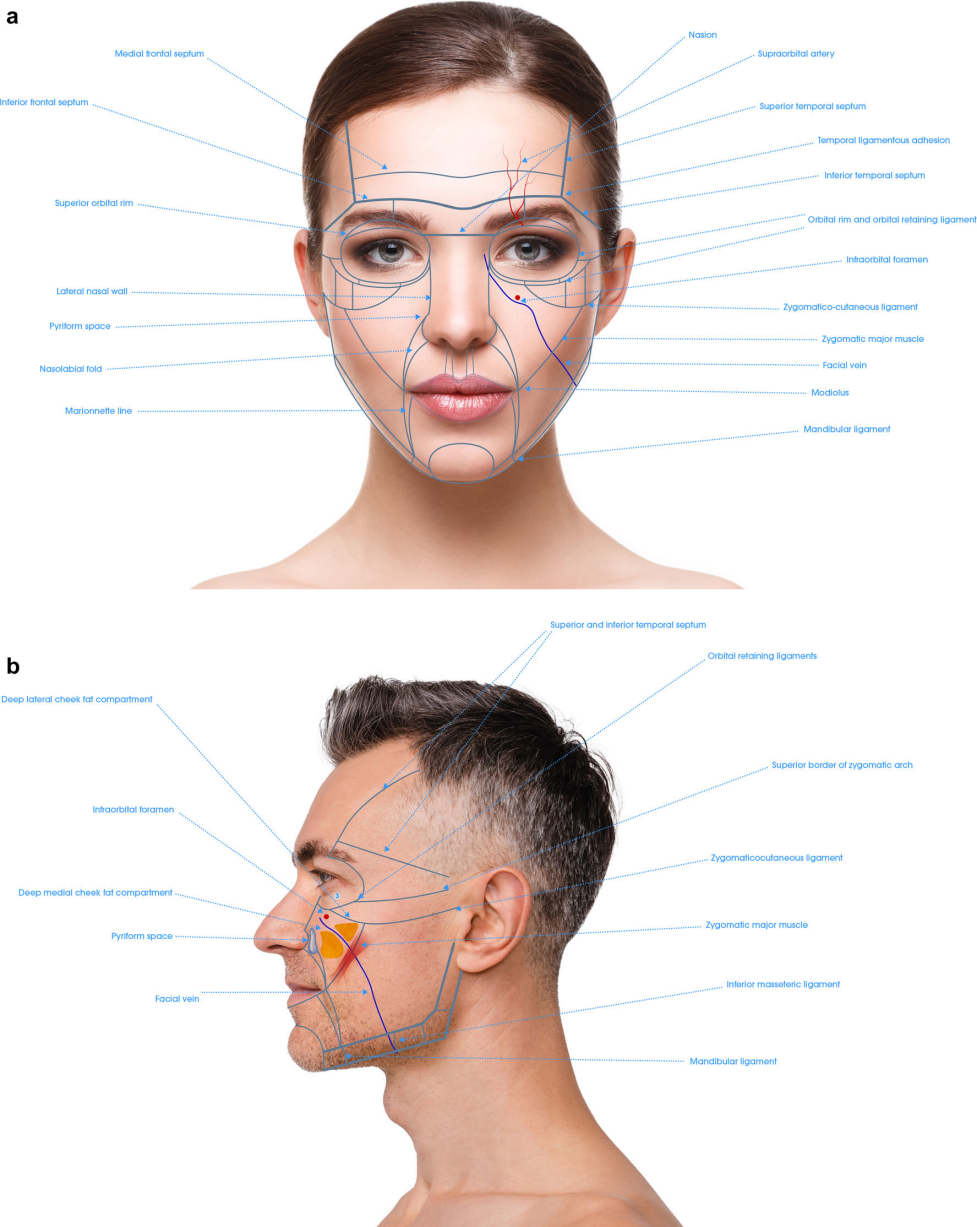

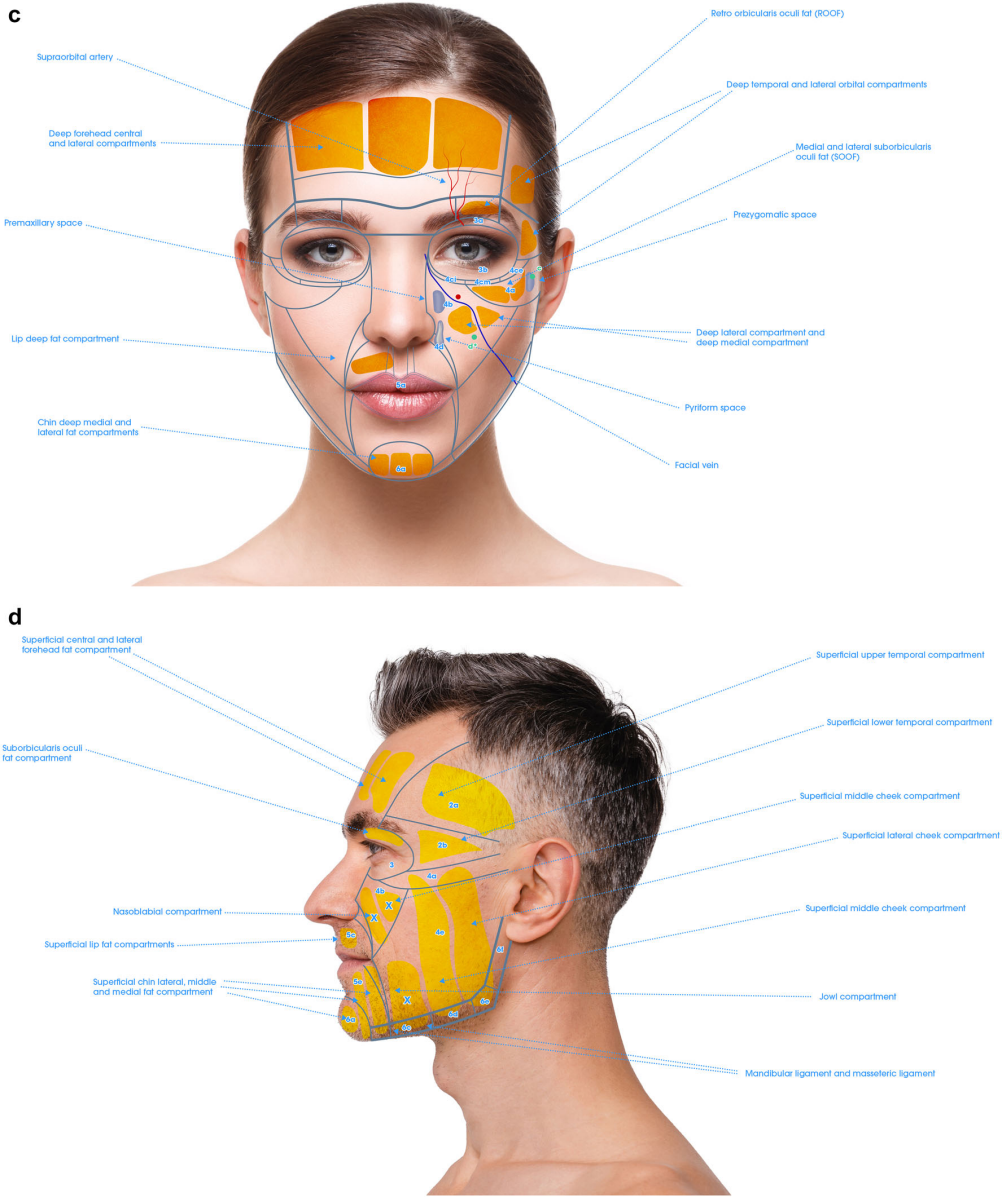
**a** Frontal view of the anatomical components of the NAUs. **b** Profile view of the anatomical components of the NAUs .**c** Frontal view of the NAUs and their associated deep fat compartments. **d** Profile view of the NAUs and their associated superficial fat compartments

Keeping these prerequisites in mind, the face can be divided in 6 NAUs (Fig. 1a, b). Each NAU has between 1 and 11 subunits which are the minimal anatomical section that should be treated entirely to obtain an aesthetic result. The anatomical background, targeted fat compartments, and facial spaces of NAUs are illustrated in Fig. 2a–d [12–14].

Another important aspect of NAUs’ subunits is that each one has a unique and constant surface curvature. In Tables 1, 2, 3, 4, 5, and 6 the ideal curvature of each NAUs’ subunit is described for Caucasian female patients. Their curvature is constant within a subunit and can be convex or concave. Between NAUs’ subunits, the curvature may vary, becoming more pronounced or reverse from concave to convex and vice versa. However, the transition between NAUs’ subunits should remain smooth.

**Table 1:** NAU 1 treatment approach

Layer treated	Curvature	Products recommended	Entry point	Device	Technique	Volume in mL	Danger
Subunit 1a Deep central and lateral forhead compartements	Convexe apex at midline at half height of the forehead	RHA2	Point A1 and A2	Cannula	Fanning	0.2–0.4	Frontal branch temporal artery, supraorbital and trochlear artery are superficial to frontal muscle
Subunit 1b Intradermal		RHA1, RHA2	Within subunit limits	Needle	Linear retrograde	0.1–0.2	Frontal branch temporal artery, supraorbital and trochlear artery are superficial to frontal muscle Cave compression of vessels by over-injection
Subunit 1c ROOF		RHA2, RHA3	Within subunit limits	Needle	Bolus	0.2–0.3	Stay lateral to supra-orbital notch
			Point B or C	Cannula	Linear retrograde	0.2–0.3	
Subunit 1d Epiperiosteal		RHA2	Within subunit limits	Needle	Bolus	0.2–0.3	Stay lateral to supra-orbital notch

**Table 2:** NAU 2 treatment approach

Layer treated	Curvature	Products recommended	Entry point	Device	Technique	Volume in mL	Danger
Subunit 2a Subperiosteal	Slightly concave with the maximum depth at the inferior temporal septum	RHA3, RHA4	Perpendicular injection	Needle	Bolus	0.2–0.4	Deep and middle temporal artery
Subunit 2a and 2b Interfascial		RHA2, RHA3	Point B or C	Cannula	Linear Retrograde	0.2–0.3	Superficial temporal artery runs in the inner layer of superficial temporal fascia, the frontal nerve runs deep to superficial temporal fascia
Subcutaneous		RHA1, RHA2	Point B or C	Cannula	Linear Retrograde	0.2–0.4

**Table 3:** NAU 3 treatment approach

Layer treated	Curvature	Products recommended	Entry point	Device	Technique	Volume in mL	Danger
Subunit 3a Submuscular	Slightly concave	RHA2	Point E	Cannula	Linear Retrograde	0.1–0.2	Supraorbital and trochlear artery are superior and deep
Subunit 3b Intradermal		Redensity 1	Within subunit limits	Needle	Micro aliquots	0.01

**Table 4:** NAU 4 treatment approach

Layer treated	Curvature	Products recommended	Entry point	Device	Technique	Volume in mL	Danger
Subunit 4ai Medial SOOF	Very convex	RHA3, RHA4, Ultra deep	Perpendicular injection	Needle	Bolus injection	0.1–0.2	Zygomaticofacial artery
			Point C	Cannula	Linear Retrograde	0.1–0.2	
Subunit 4ai superficial compartments Medial infraorbital fat compartment		RHA3, RHA4	Point C	Cannula	Linear Retrograde	0.1–0.2	Zygomaticofacial artery, transverse facial artery
Subunit 4am deep compartments Lateral SOOF		RHA3, RHA4, Ultra Deep	Perpendicular injection	Needle	Bolus injection	0.1–0.2	Zygomaticofacial artery, transverse facial artery
			Point C and D	Cannula	Linear Retrograde	0.1–0.2	
Subunit 4am superficial compartments Lateral infraorbital fat compartment		RHA3, RHA4	Point C	Cannula	Linear Retrograde	0.1–0.2	Zygomaticofacial artery, transverse facial artery
Subunit 4ae Prezygomatic space		RHA3, RHA4, Ultra deep	Perpendicular injection	Needle	Bolus injection	0.1–0.2	Zygomaticofacial artery, transverse facial artery
			Point C and D	Cannula	Linear Retrograde	0.1–0.2	
Subunit 4ae superficial Lateral and medial fat compartment of the cheek		RHA3, RHA4	Point C	Cannula	Linear Retrograde	0.1–0.2	Zygomaticofacial artery, transverse facial artery
Subunit 4bi Premaxillary space	Convex	RHA3, RHA4, Ultra Deep	Perpendicular injection	Needle	Bolus injection	0.1–0.3	Facial artery, infraorbital artery
			Point D	Cannula	Linear Retrograde	0.1–0.3	
Subunit 4bm Deep medial fat compartment		RHA3, RHA4, Ultra deep	Perpendicular injection	Needle	Bolus injection	0.1–0.3	Facial artery, infraorbital artery
			Point C and D	Cannula	Linear Retrograde	0.1–0.3	
Subunit 4ae Deep lateral fat compartment	Slightly concave	RHA3, RHA4, Ultra deep	Points C and D	Cannula	Linear Retrograde	0.1–0.2	Facial artery, infraorbital artery
Subunit 4ci Tear through ligament		Redensity 2	Point D	Cannula	Linear Retrograde	0.1–0.25	Angular artery
Subunit 4cm Medial tear through, subperiosteal		Redensity 2	Points C and D	Cannula	Linear Retrograde	0.1–0.25	Zygomaticofacial artery
Subunit 4ce Subperiosteal lateral orbital rim		Redensity 2	Points C and D	Cannula	Linear Retrograde	0.1–0.25	
Subunit 4d Superficial medial and lateral fat compartments		RHA3, RHA4	Points C and D	Cannula	Linear Retrograde	0.25–1	
Subunit 4e Nasolabial fold dermis		RHA1, RHA2, RHA3, RHA4, Ultra deep	Within subunit limits	Needle	Linear Retrograde	0.2–0.4	Facial artery, superior labial artery
Subunit 4e Pyriform space		RHA4, Ultra deep	Within subunit limits	Needle	Gun shot technique	0.1–0.25

**Table 5:** NAU 5 treatment approach

Layer treated	Curvature	Products recommended	Entry point	Device	Technique	Volume in mL	Danger
Subunit 5a Vermilion border	Line	RHA1, RHA2, RHA3	Along vermilion border	Needle	Linear Retrograde	0.05	Superior labial artery
Subunit 5a Vermillion body	Convex	RHA1, RHA2, RHA3	Vermillion body	Needle	Linear Retrograde	0.05–0.15	
Subunit 5a Russian technique		RHA2, RHA3	Perpendicular to vermillion border	Needle	Linear Retrograde vertical	0.01 per pass	Superior labial artery
Subunit 5b Vermilion border	Line	RHA1, RHA2, RHA3	Along vermilion border	Needle	Linear Retrograde	0.05	Inferior labial artery
Subunit 5b Vermillion body	Convex	RHA2, RHA3	Vermillion body	Needle	Linear Retrograde	0.01 per pass	
Subunit 5b Russian technique		RHA2, RHA3	Perpendicular to vermillion border	Needle	Linear Retrograde	0.01 per pass	
Subunit 5c Philtrum	Concave with two lines laterally	RHA1, RHA2, RHA3	Vermilion border	Needle	Linear Retrograde	0.05	Columellar arteries
Subunit 5c Lateral aspect	Slightly concave	RHA1	Within subunit limits	Needle	Linear Retrograde		
Subunit 5ds Dermis		RHA1, RHA2, RHA3	Point E	Needle	Linear Retrograde	0.05–0.15	Facial artery, superior labial artery
Subunit 5ds Subcutaneous		RHA3, RHA4		Cannula	Linear Retrograde	0.1–0.2	Transverse mental artery
Subunit 5di Dermis		RHA1, RHA2, RHA3		Needle	Linear Retrograde	0.05–0.15	Facial artery, superior labial artery
Subunit 5di Subcutaneous		RHA3, RHA4		Cannula	Linear Retrograde	0.1–0.2	
Subunit 5e Subcutaneous		RHA3, RHA4		Cannula	Linear Retrograde	0.2–0.5	Transverse mental artery

**Table 6:** NAU 6 treatment approach

Layer treated	Curvature	Products recommended	Entry point	Device	Technique	Volume in mL	Danger
Subunit 6a Deep mental fat compartment	Very convex	Ultra Deep	Perpendicular median and 2 lateral	Needle	Bolus	0.01–0.8	Ascending mental artery
Subunit 6a Superficial mental fat compartment		RHA3, RHA4	Point E	Cannula	Linear Retrograde	0.05–0.15	
Subunit 6b Lateral superficial mental fat compartment	Line	RHA3, RHA4	Point G, point F, point E	Cannula	Linear Retrograde	0.05–0.25	Submental artery and ascending branches
Subunit 6c Jowl fat compartment Only in young patient		RHA4	Point G, point F, point E	Cannula	Linear Retrograde	0.05–0.3	
Subunit 6d Medial superficial fat compartment of the cheek		RHA4	Point G, point F, point E	Needle	Linear Retrograde	0.05–0.03	Columellar arteries
Subunit 6e Periosteum of mandibular angle		Ultra Deep	Perpendicular	Needle	Bolus	0.01–0.3	Parotid gland, masseter muscle
Subunit 6f Lateral superficial fat compartment of the cheek		RHA4	Point G	Cannula	Linear Retrograde	0.05–0.15	Facial artery, superior labial artery

In four areas, the NAUs’ subunit transition zone is an abrupt change of curvature that creates a line: jawline, vermilion border of upper and lower lips, and philtrum.

NAU 1 is convex, its apex is usually situated at midline at the half of its height. NAU 2 is slightly concave, and its deepest portion corresponds to the inferior temporal septum. NAU 3a and NAU 3b are slightly concave with curvature changing to convex in neighboring NAU 1b, NAU 1c superiorly, and NAU 4c inferiorly. NAU 4a is very convex, NAU 4b is convex, NAU 4c is slightly concave and NAU 4d is slightly concave. Between NAU 4b and NAU 4d, the curvature changes from slightly convex to slightly concave.

NAU 5a and NAU 5b are concave with an abrupt change of curve at their junction with respectively NAU 5c and NAU 5d, which are concave. NAU 6a is convex. NAU 6b to NAU 6f are slightly convex with a smooth change of curve at junction with NAU 4d and NAU 5d, and abrupt change of curve with the neck that creates a line.

### Step 1: Assessment

Using NAUs, a two-step process involving assessment and treatment is adopted, enabling the creation of a personalized plan. Naturally, a complete medical history should exclude patients that are not eligible for HA fillers treatment.

Correction of soft tissue and bone volume deficiency are key elements in rejuvenation and beautification of the face with HA fillers [15]. When evaluating volume, asymmetry must also be assessed, as it leads to a significant overall improvement [16, 17]. Each NAU is evaluated for 3 degrees of volume deficiency: none (N), moderate (M) and severe (S). The NAUs with the highest scores should be considered first for treatment. This decision is obviously discussed with the patient and their preference should be taken into consideration (Figs. 7, 8).

Beside volume deficiency and asymmetry, two other elements should be analyzed: transition between NAUs’ subunits and sagging of soft tissue. The transition between NAUs’ subunits, corresponding to the border between two NAUs, should be concealed in most cases. The exceptions to this rule are the vermillion and philtrum borders (NAU 5) and jawlines (NAU 6), where transition is abrupt and creates an edgy aspect. The last element to be considered is sagging of soft tissue, which is indirectly addressed by volume restoration. There are several areas of sagging that can be addressed: eyebrows (NAU 1c), cheeks-nasolabial fold junction (NAU 4d) and jowls (NAU 6c). Their treatment modalities are explained in the corresponding treatment section below.

### Step 2: Treatment

**Fig. 3:**
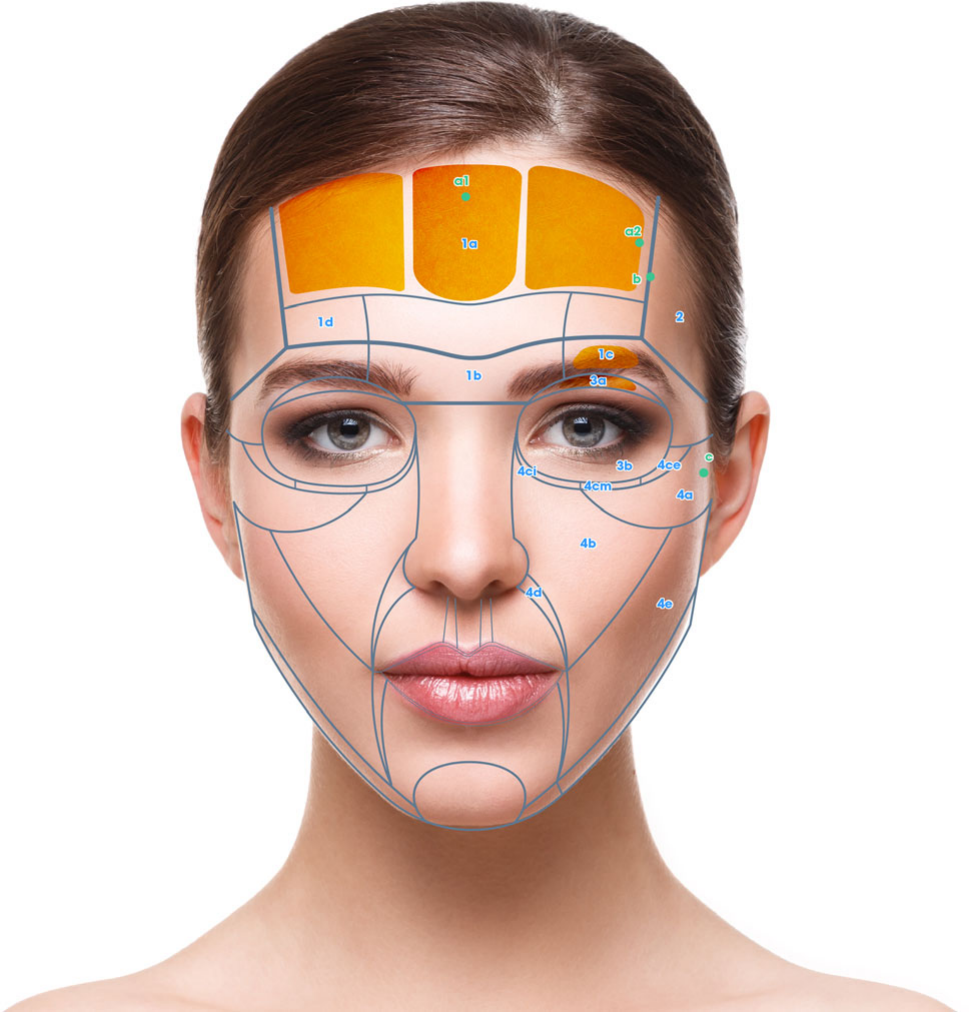
Frontal view of NAU 1 and NAU 3, and respective subunits. Entry points a1, a2, b, c are represented in green

**Fig. 4:**
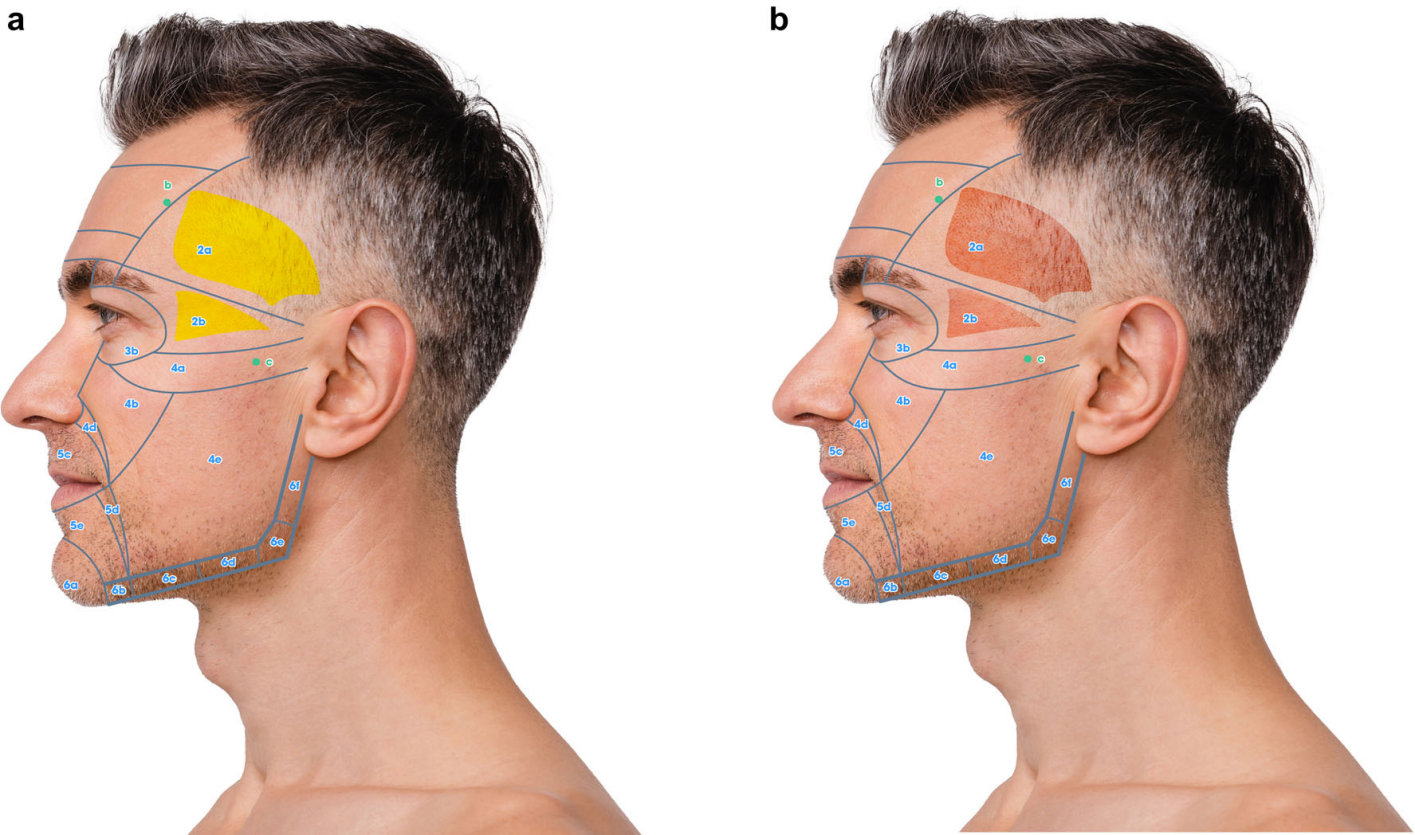
**a** Frontal view of the superficial fat compartments associated with NAU 2 Entry points b and c are represented in green. **b** Frontal view of the interfascial compartments associated with NAU 2. Entry points b and c are represented in green.

**Fig. 5:**
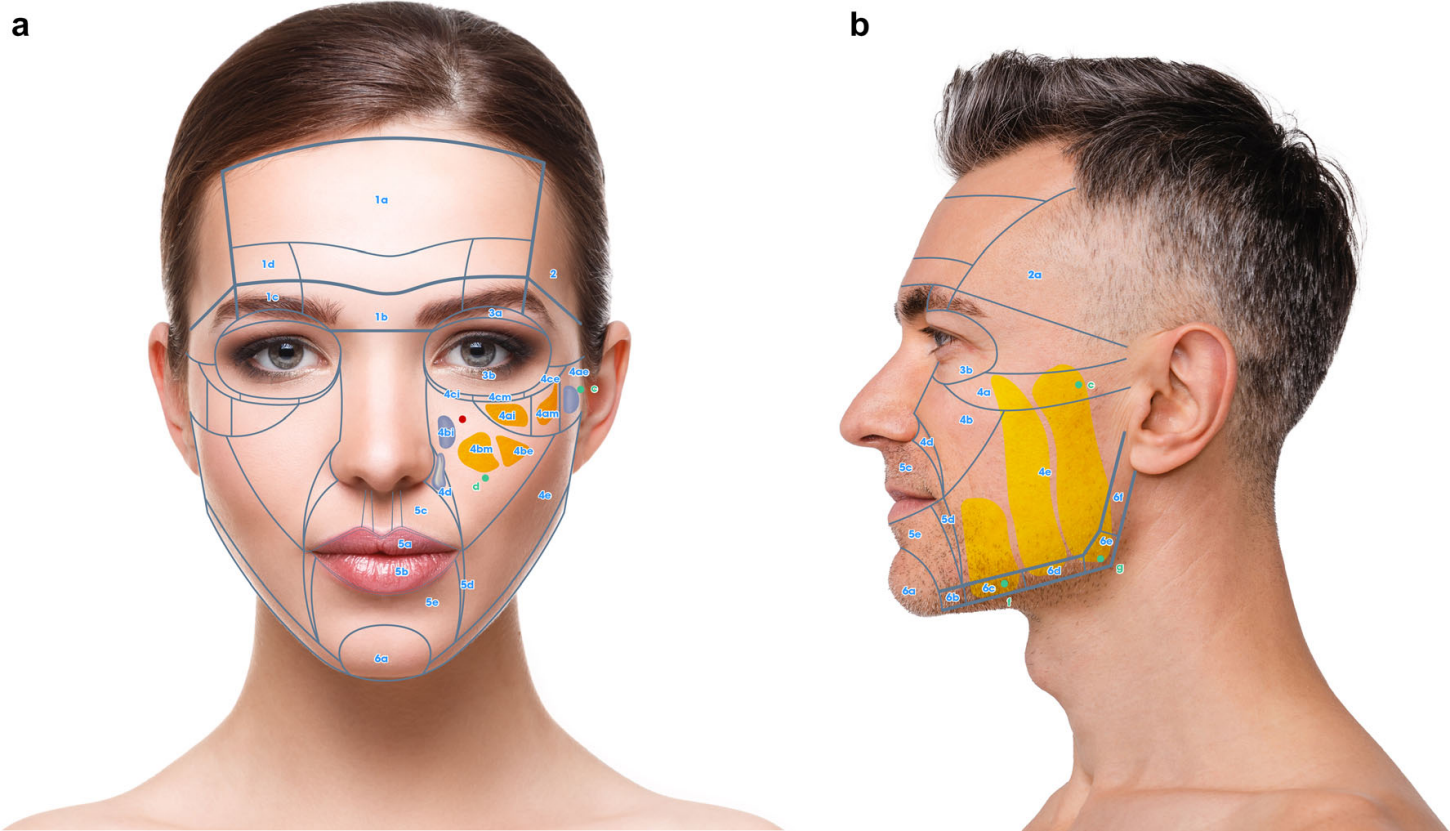
**a** Frontal view of subunits NAU 4a, 4b, 4c and 4d. Entry points c and d are represented in green. **b** Profile view of subunits NAU 4e, 6c, 6d, 6e, 6f and associated superficial fat pad compartments. Entry point e is represented in green

**Fig. 6:**
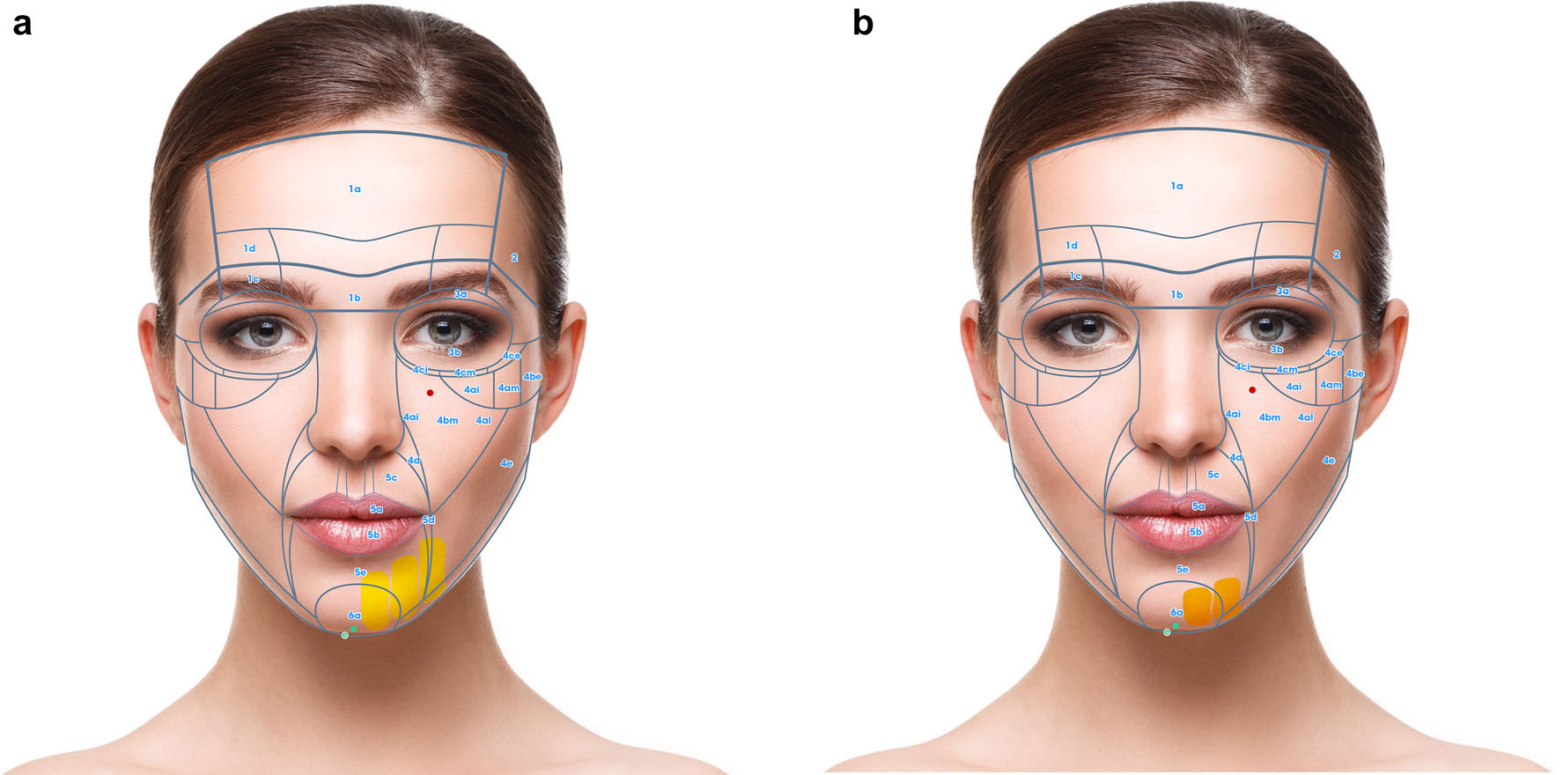
**a** Frontal view of NAU 5, NAU 6a and 6b and associated superficial fat pad compartments. Entry points c, f and g are represented in green. **b** Frontal view of subunits NAU 6a and 6b and associated deep fat pad compartments. Entry point e is represented in green

For each NAU and respective subunits the treatment technique is illustrated in Figs. 3, 4, 5, and 6 and described in Tables 1, 2, 3, 4, 5, and 6. For each NAU subunit, the precise targeted structure is described, including superficial fat compartments, facial spaces, deep fat compartments, and dermis. The boundaries, as well as the name of fat compartments and facial spaces are based on the description by Cotofana et al. [14].

Treating from a cranial to caudal and lateral to medial direction can achieve an indirect but significant improvement and lifting effect of neighboring inferior and medial NAUs [18].

The only exception is the treatment of tear troughs (NAU 4ci, 4cm and 4ce). In this case, the NAUs 4a and 4b should be treated first. Hence, addressing NAU 4a and 4b first leads to an improvement of the axis and the depth of the tear trough and decreases the volume needed to treat NAU 4ci, 4cm and 4ce.

In Tables 1, 2, 3, 4, 5, and 6 the following treatment’s recommendations are provided [9, 19, 20].

- Precise targeted structure and layer(s).
- Curvature.
- Best device (cannula vs. needle).
- Entry point(s) for cannulas.
- Best technique(s) of injection.
- Name of products used and quantity: the Teoxane (Teoxane SA, Geneva, Switzerland) range of products was used as it is adapted for multilayering technique [10]. The selection of products is based on the author’s routine clinical practice. Nonetheless, the author considers this tool a valuable instrument for defining treatment plans with other HA-based dermal fillers that have similar characteristics.
- Danger zones.

## NAUs’ Unit by Unit Treatment Modalities

### NAU 1: Forehead

Forehead has a complex anatomy [21]. In the NAU 1a and 1b the deep fat compartments that has a vertical axis are targeted. This injection improves the contour of the forehead and has an indirect lifting effect on the eyebrows. Moreover, Subunit 1c, which corresponds to the retro orbicularis oculi fat (ROOF) is also targeted and has a lifting effect on the apex and the tail of the eyebrow. In case of local depression, NAU 1d can be treated on the periosteum (Fig. 3 and Table 1). Finally, for optimal eyebrow reshaping, injection of NAU 2 is also helpful. The danger zones correspond to the supratrochlear and supraorbital arteries, which are deep to the frontal muscle above the orbital rim and become superficial cranially. They are directly connected to the ophthalmic artery. HA fillers injection in the forehead must be very precise to avoid any vessels that run deep to the frontalis muscle below the inferior frontal septum, and on top of the frontalis muscle above the medial frontalis septum (Fig. 2).

### NAU 2: Temporal Area

To treat NAU 2 in PFAC, three different layers can be targeted with HA fillers: on the periosteum in the upper part of temporal fossa, between superficial and deep temporal fascia (interfascial), and in the subcutaneous layer [22]. Details of the treatment modalities are described in Fig. 5 and Table 2.

According to the author, injection on the periosteum is safer above the inferior temporal septum (NAU 2a), where temporal fossa is shallower. This area presents a lower risk of accidental injection in the deep temporal artery and the deep temporal fat compartment (in continuity with the boule de Bichat). Concerning both NAU 2a and 2b, interfascial and subcutaneous injection can be applied [9, 23]. There is evidence suggesting that epi-periosteal injection has the higher effect on temporal volume correction and temporal crest hiding, that interfascial injection improves eyebrow position and crow’s feet, and that subdermal injection has a lifting effect on NAU 4 and NAU 6 [9, 24].

### NAU 3: Orbital Area

In this unit, skin is very thin and adherent to the orbicularis muscle. By using a cannula at the entry point B, the submuscular layer in contact with the periosteum of the superior orbital rim is targeted. Concerning NAU 3b, the sole recommended treatment is dermal injection with a non-reticulated product to improve skin quality of the crow’s feet. Treatment of NAU 3a and 3b should be very parsimonious as swelling and visibility is a major problem. Details of the treatment modalities are described in Fig. 3 and Table 3.

### NAU 4: Cheek Area

Details of treatment modalities are described in Fig. 5 and Table 4. For NAU 4a, targeted compartments are the medial suborbicularis oculi fat compartment (medial SOOF, or NAU 4bi), lateral suborbicularis oculi fat compartment (lateral SOOF, or 4bm) and the lateral aspect of pre-zygomatic space, which we call NAU 4be [25]. The SOOFs and prezygomatic space augmentation permits a lifting effect of the cheek, whereas the augmentation of the deep medial cheek (DMC, or NAU 4am) and deep lateral cheek (DLC, or NAU 4al) will increase projection of the cheek [26]. The NAU 4e injection seems to have the highest rate of correction per volume of HA fillers injected among all deep fat compartments of the NAU 4 [27]. The injection is done in contact with the periosteum of the zygomatic arch.

Superficial fat compartments composing the NAU 4a are: the medial infraorbital fat compartment, lateral infraorbital fat compartment and the superior aspect of medial and lateral superficial fat compartments of the cheek (Fig. 5 and Table 4). The medial infraorbital fat compartment has a weak lymphatic drainage, and any trauma can induce swelling, leading to malar edema. Hence, avoiding injection of this fat compartment, especially if there is a sign of preexisting malar edema, is recommended. In case of malar edema, the author advises to use a cannula since, by remaining deep to the infraorbital fat compartment, one can avoid triggering malar edema and still treat the deep compartment of NAU 4.

For NAU 4b, the DLC fat compartment seems to have the most important impact on cheek projection and the correction of the V deformity [15]. Besides the medial and lateral fat compartments, the maxillary space (i.e., virtual space situated between orbicularis oculi muscle and labii superioris alaeque nasi) can also be treated. Care should be taken to avoid the facial artery in this area, which is medial to maxillary space and runs in subcutaneously above the level of the alar base, as well as the infraorbital artery that is medial to the maxillary space (Fig. 5 and Table 4).

The middle superficial fat compartment (i.e., NAU 4b) should be avoided because of the risk of caudal displacement of HA filler in this mobile, moderately large and vertical fat compartment [7]. It is also recommended to avoid injection of the nasolabial fat compartment (NAU 4d) and jowl fat compartment (NAU 6c), as this will aggravate aging signs.

Concerning NAU 4a and 4b, there are two techniques of treatment: (1) bolus injection with needle targeting individually each compartment; and (2) Linear retrograde injection using a cannula at entry points C and D (Fig. 5a).

In NAU 4c (Fig. 5a), the targeted structures are the tear trough ligaments and the palpebromalar groove. The first is a unique ligament between the bone and the skin. The latter is a multilamellar ligament with a weaker attachment to the skin. The injection should be deep in contact with the periosteum. In the tear trough, it’s advisable to administer a minimal volume of injection. This precaution is necessary as it seems that HA fillers have a tendency to migrate to the superficial layer through the orbicularis muscle, potentially leading to palpebral swelling [28, 29]. Due to proximity of the angular artery and the tendency for bruising, the use of cannulas is recommended to treat this area. The importance of blending the lid-cheek junction (between NAU 4b and 4c) has already been described in surgery literature [30], prompting the use of HA fillers to significantly enhance this area. Finally, to optimize the result and conceal boundaries of NAU 4ce (palpebromalar groove), treatment should be performed beyond the lateral limit of the former, at the external canthus and in contact with the zygoma’s periosteum. This will conceal the boundary between NAU 4ce and NAU 2.

When addressing NAU 4c, a “triangulation technique” is used. This technique is very useful when the area is dangerous to inject, or a very precise injection is required. The index fingertip of the non-dominant hand is placed on the orbital rim exactly at the injection target, and the cannula is advanced until contact is also reached.

Finally, if 4a, 4b and 4c should be treated together, 4a and 4b are to be treated first. Treating the deep cheek compartments appears to lift the area and provide support to the orbital retaining ligament (ORL) and zygomatico-cutaneous ligament (ZCL) which improves 4c [31]. In some mild cases, treating NAU 4a may be sufficient, eliminating the need for further treatment of NAU 4c. Before deciding to treat NAU 4c, some principles are to be respected. In case of severe sagging of inferior eyelid and/or fat pockets, treatment of NAU 4b and 4c should be carefully evaluated [32] and potential results discussed with the patients.

As there are no deep fat compartments in NAU 4d, medial and lateral superficial fat compartments are the injection targets due to their large surfaces. The author recommends to inject using a cannula with a fanning technique, from entry point C and E, where the skin is less mobile. This facilitates the injection process, thanks to the support provided by the zygomatico-cutaneous ligament and mandibular septum, respectively. These ligaments also help to avoid migration of HA fillers cranially and caudally, respectively. In some cases, there is a need to blend the border between subunits using the superficial fat compartments. Hence, the junction between medial and middle fat compartment on the border between 4a and 4d, as well as the superior aspect of the middle and lateral fat compartment that traverse the border between 4b and 4d, are targeted.

NAU 4e corresponds to the nasolabial fold (NLF) (Fig. 5e). The treatment techniques for this area should avoid the facial artery and its branches. The safest layer is the thick dermis or immediate subdermal layer, which can be targeted with a needle. The author’s recommended treatment of 4e is a multilayering technique targeting dermis and the pyriform space with needle [7]. The treatment of this unit is preferably performed after NAU 4a, 4b and 4c, which would decrease the depth of the NLF by a lifting effect.

### NAU 5: Oral Area

NAU 5 (Fig. 6a) corresponds to the oral area, which shows similar characteristics to palpebral areas, namely no posterior attachment to bone, and very mobile and thin, soft tissue layers. Injection of the oral area should be precise and conservative to cope with its dynamic aspect. Another particularity is the extensive arterial network and the inherent risk of skin necrosis. The labial arteries, which in most cases run submucosally, are avoided with a subcutaneous injection. Columellar arteries run in the subcutaneous layer, thus injection of philtrum and upper white lips should be performed in the dermis. The transverse mental arteries, which run under the superficial musculo-aponeurotic system (SMAS), are avoided by a subcutaneous injection in NAU 5e. Volume restoration of the lips is performed intradermally in the white lips and subcutaneously in the red lips to decrease the risk of vascular complications. The aim of HA fillers is to improve lips volume, shape, projection and decrease vertical lines in mature patients. Details of treatment details are described in Fig. 6a and Table 5.

### NAU 6: Chin and Jawlines

NAU 6 (Figs. 5b, 6a, b) can be compared to a pyramid where the tip is located at the chin and the two bases at the gonial angles. The treatment is usually started in these three points to create a skin tension line between them. Next, the injector can treat the soft tissue “gap” along jawlines with HA fillers.

Generally, in NAU 6a, the augmentation area with HA fillers corresponds to the mouth width in male and nasal base width in female patients. This area features an abundant vascular network. Ascending mental arteries run deep in the muscle or in contact with the periosteum. Additionally, there is an anastomosis between the submental and sublingual arteries. Along the jawline, the facial artery and vein are in the subplatysmal layer medial to the masseter muscle and should be avoided. In older patients with jowls, treating NAU 6c situated between masseter and mandibular ligament should also be avoided to prevent worsening of the phenomenon [33]. The author recommends treating jowls by injecting NAU 6a and 6e bilaterally on the periosteum with very cohesive HA fillers. In a second step, NAU 6b, 6d, 6e and 6f should be treated subcutaneously while avoiding area 6c (i.e., jowls), especially in more mature patients, which will improve the contour and conceal of the latter. To obtain a well-defined facial definition in the periauricular area (NAU 6f) the subcutaneous fat is treated. Details of treatment modalities are described in Fig. 5b and Table 6.

NAU 6 has a thin, soft tissue layer and two powerful ligaments (i.e., mandibular and masseteric ligaments). Treatment with HA dermal fillers can lead to well defined jawlines. Moreover, the treatment will also induce a lifting effect on the neck soft tissue.

### Step 3: Planning

The personalized treatment plan for two patients based on the application of the NAU method is recorded in Tables 7 and 8. Patient’s priorities and desires should naturally be taken into consideration and can influence the modification of the treatment plan. This plan is a helpful tool to establish a diagnostic map, facilitate communication between patients and the healthcare team, data statistical analysis.

**Table 7:** Treatment plan for patient 1

Dates	NAUs’ subunits	Score	Products	Device/entry point	Technique	Product quantity left/right side in mL
Day 0	4ai	M	RHA4	27 G needle	Bolus	0.15/0.15
	4am	M	RHA4	27 G needle	Bolus	0.15/0.15
	4ae	M	RHA4	27 G needle	Bolus	0.1/0.1
	4bm	M	RHA4	27 G needle	Bolus	0.15/0.15
	4be	M	RHA4	27 G needle	Bolus	0.15/0.15
	4d	M	Ultra Deep	27 G needle	Bolus	0.2/0.2
	4ci	M	Redensity 2	Cannula/Point D	Linear retrograde	0.15/0.15
	4 cm, 4ce	M	Redensity 2	Cannula/Point C	Linear retrograde	0.3/0.3
	4a	M	RHA3	30G	Needle	0.3/0.3
	4b	M	RHA3	30G	Needle	0.2/0.2
	5ds and 5di intradermal	M	RHA3	30 G needle	Linear retrograde	0.2/0.2
	5di subcutaneous, 5e, 6a superficial	M	RHA4	Cannula/Point E	Linear retrograde	0.5/0.5
	6a	M	Ultra Deep	27 G needle	Bolus	0.6

**Table 8:** Treatment plan for patient 2

Dates	NAUs’ subunits	Score	Products	Device/Entry point	Technique	Product quantity left/right side in mL
Day 0	6a deep	M	Ultra Deep	27 G needle	Bolus	0.6
	5di subcutaneous, 5e, 6a superficial	M	RHA4	Cannula/Point E	Linear retrograde	0.6
	6e	M	Ultra Deep	27 G needle	Bolus	0.3/0.3
	6b,6d,6e and 6f	M	RHA4	Cannula/Point G	Linear retrograde	1.2/1.2
Day 20	6e deep	M	Ultra Deep	27 G needle	Bolus	0.2
	6b, 6d, 6e	M	RHA4	Cannula/Point G	Linear retrograde	0.3:0.3

**Fig. 7:**
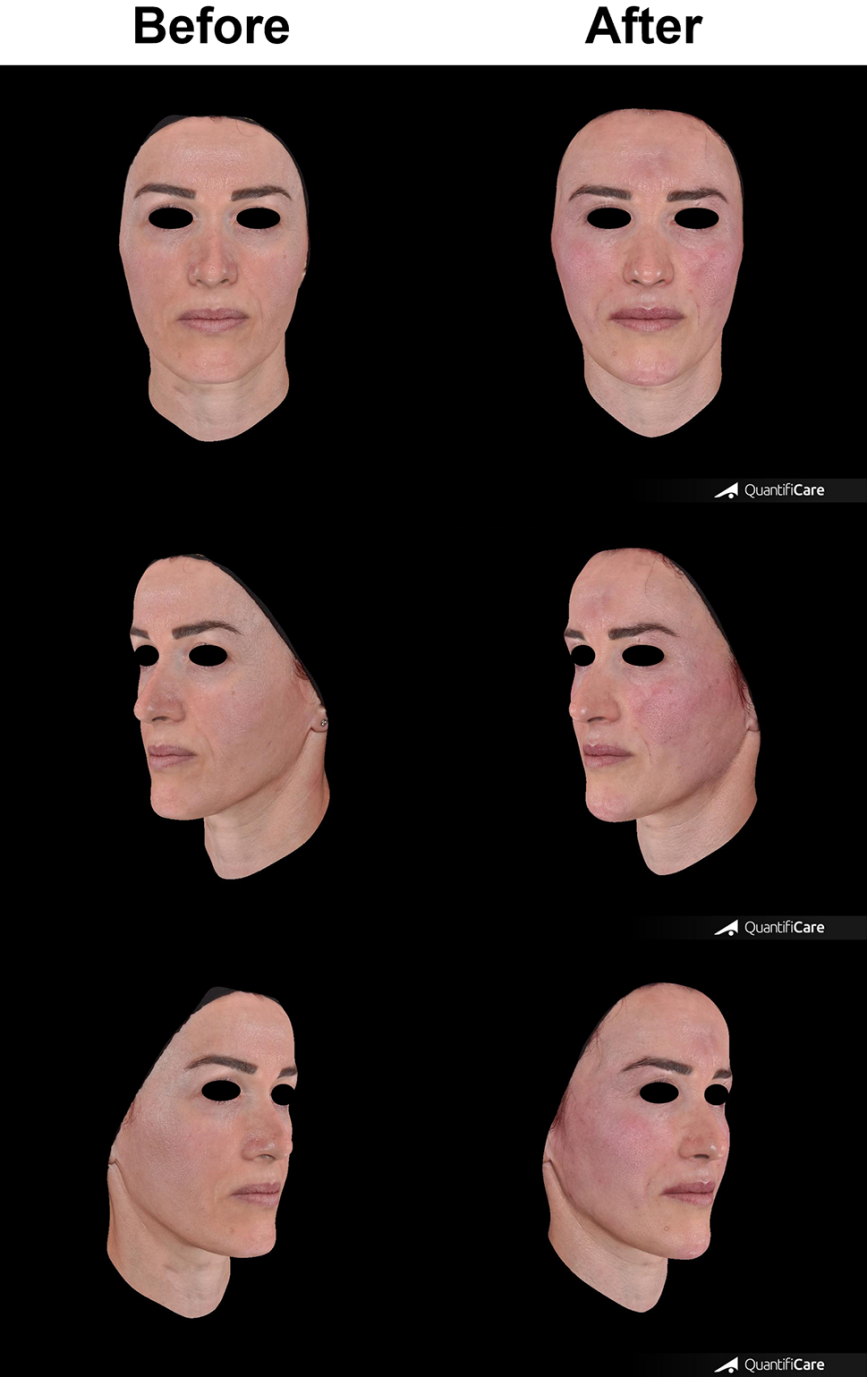
Before-and-after pictures of Patient 1 following full-face treatment: RHA3 (2 mL), RHA2 (1 mL), RHA4 (3 mL), and Ultra Deep (1.5 mL)

**Fig. 8:**
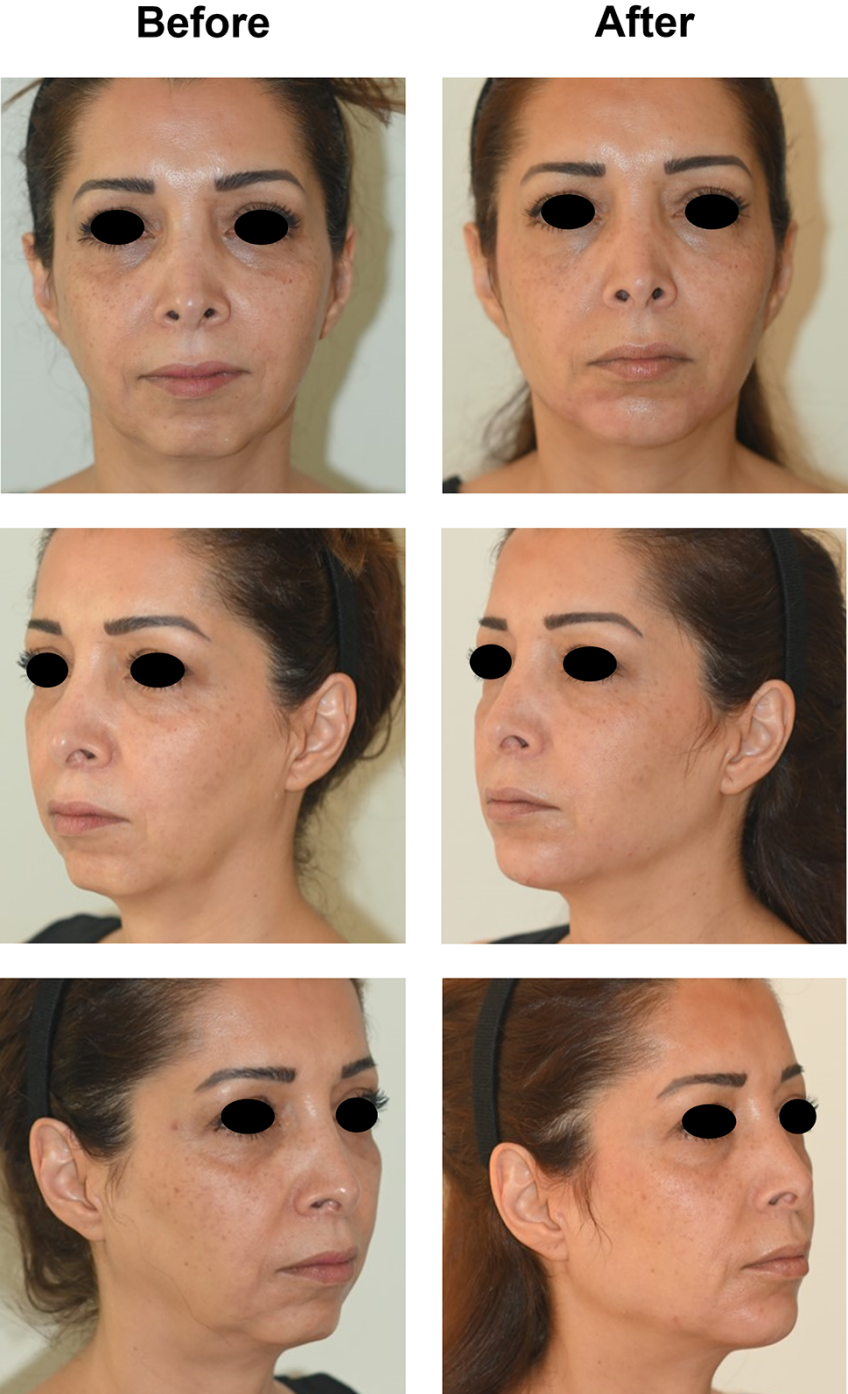
Before-and-after pictures of Patient 2 after NAU 6 treatment: NAU 6a (0.6 mL Ultra Deep subperiosteal), Ultra Deep (2 mL), and RHA4 (2.5 mL)

The treatment plan also encompasses the number of steps and their timing (Tables 7, 8**)**. Thus, depending on the number of NAUs to be treated, the treatment can be organized in one or several sessions. The interval between two sessions is typically 3–4 weeks, allowing for the post-injection swelling from the previous session to subside. Figures 7 and 8 depict before and after photographs of two patients treated with the multilayering and composite techniques.

## Discussion

The NAU method is a step by step, precise and practical system for the assessment and treatment of PFAC with HA fillers. In this method, the face is divided into 6 different new aesthetic units (NAU), which have between 2 and 11 subunits. The design of NAUs’ subunits corresponds to the minimal facial section to be treated to achieve an aesthetic outcome. In general, treatment of an entire NAU is the best option for optimal aesthetic results.

In the first step (i.e., assessment), volume deficiency is analyzed and recorded for each NAUs’ subunits. Hence, a severity score from no deficiency to severe deficiency is established for each NAUs’ subunit providing a ranking. The NAUs with the severest volume deficiency should be considered first for treatment. In the second step (i.e., treatment), the anatomical structures to be targeted are precisely described for each NAU (Figs. 3, 6). These structures include deep fat compartments, superficial fat compartments, facial spaces, and dermis. In some subunits, a monolayer treatment is suggested, in others a multilayer treatment is preferred. These recommendations are based on best practice medicine supported by large international expert consensus [8, 9].

Lastly, for each patient a personalized and precise treatment plan is established (Tables 7, 8). Naturally, the desire of the patient should also be taken into consideration. Consequently, the sequence of NAU treatment, along with the number and the timing of each step are documented. This recorded data serves as a valuable tool for facilitating communication between patients and the healthcare team, as well as for data analysis purposes. The fundamental aspects of the NAU method are that it is based on:

1. Detailed clinical anatomical knowledge, which render the assessment and treatment more accurate, facilitates beginners training and the communication with patients.
2. Structured and systematic assessment tool to analyze PFAC volume deficiency and asymmetry, which are recordable and reproducible.
3. The use of best practice medicine treatment with HA fillers by means of composite multilayering techniques. This is based on the recent progress made in the clinical anatomy and in HA fillers rheology with a large range of products adapted to different facial layers [7–9].

The NAU method is different from previously published tools [6]. The author aim was to provide anatomical landmarks for each NAU, thereby simplifying and clarifying the treatment process. The NAU method, along with the scoring tool, combines precise assessment and treatment plans, offering a straightforward approach, particularly beneficial for beginners in patient treatment. Moreover, it serves as a practical tool for communication and education purposes.

The NAU method shows several weaknesses that merit consideration. Firstly, the scoring system used in the diagnostic step is subjectively divided into three groups, which may introduce bias. Future research should aim to develop more objective scoring criteria. Additionally, while the integration of computerized 3D photography to measure volumes accurately could be beneficial [6], the high cost of these tools limits their widespread use among practitioners. In addressing volume deficiencies in PFAC, more precise determination of the layer involved, between dermis, superficial fat, deep fat, and bone, is needed. Ultrasonography has been proposed as a potentially useful tool in this regard [34]. Furthermore, given the dynamic nature of clinical anatomy in relation to PFAC, ongoing research may lead to advancements that refine or modify the NAU methodology. Lastly, this article does not address gender differences, age variations, or the nose as a distinct NAU. Additionally, NAU 5 warrants further analysis in future studies.

## Conclusion

The aim of the NAU method is to offer a comprehensive, pedagogic as well as an accurate instrument for assessment and treatment of patients with facial aesthetic complaints using HA fillers. The goal of this panfacial method, based on precise anatomy, is the precise correction of volume deficiency, asymmetry, and establishment of harmony between different units of the face. It was designed to be practical to use. Moreover, it enables communication, recording data concerning the diagnostic and the treatment plan.
